# Hellish Buttocks: The Dark Side of AQUAfilling

**DOI:** 10.5334/jbsr.3783

**Published:** 2024-12-26

**Authors:** Bluette Delire, Cristina Anca Dragean

**Affiliations:** 1 Department of Radiology, Institut de Recherche Expérimentale et Clinique (IREC), Cliniques Universitaires Saint Luc, Université Catholique de Louvain, Brussels, Belgium

**Keywords:** AQUAfilling injection, complications, MRI, CT scan

## Abstract

*Teaching point:* To highlight the potential complications associated with AQUAfilling injections, emphasizing the importance of early detection and proper management.

## Patient History

A 39‑year‑old female patient presented spontaneously to the emergency department with debilitating pain in the left buttock that had developed the previous day following intense physical exertion. Her medical history was unremarkable except for cosmetic injections of AQUAfilling in both buttocks 9 years ago. No immediate or late complications to this procedure were mentioned.

## Clinical Examination

Physical examination revealed an asymmetric swelling of the left buttock with mild erythema.

## Laboratory Findings

Significant inflammatory syndrome was noted.

## Imaging Studies

**Figure 1:**
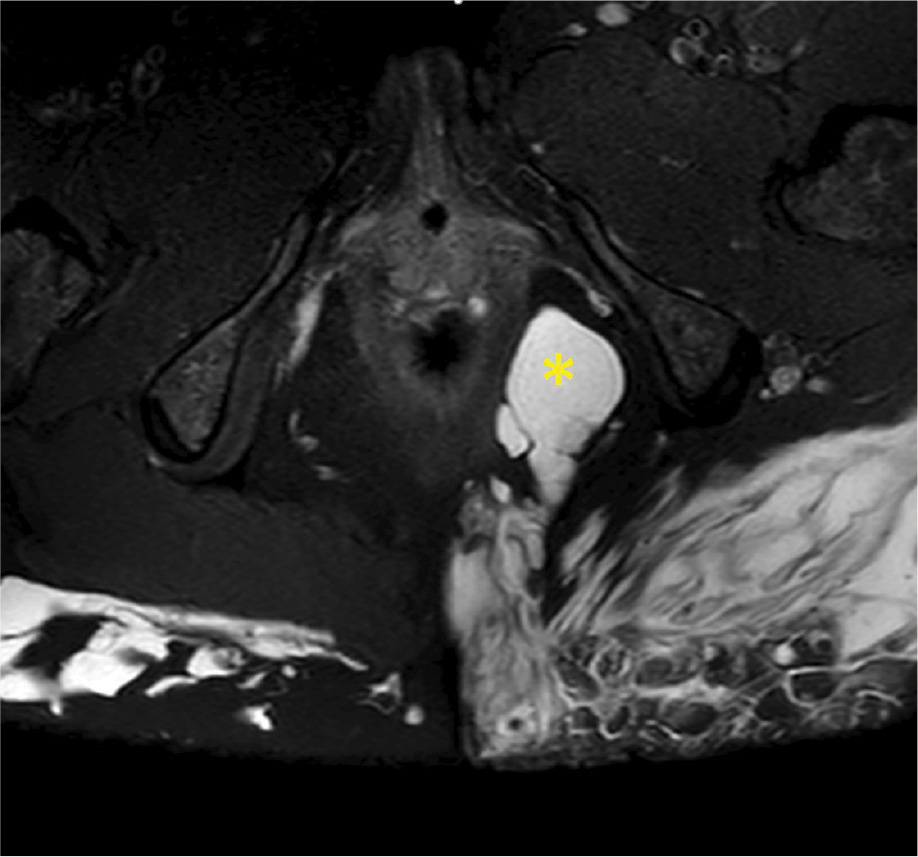
Multiloculary collections with extension in the ischio‑anal fossa and pararectal space (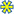).

**Figure 2:**
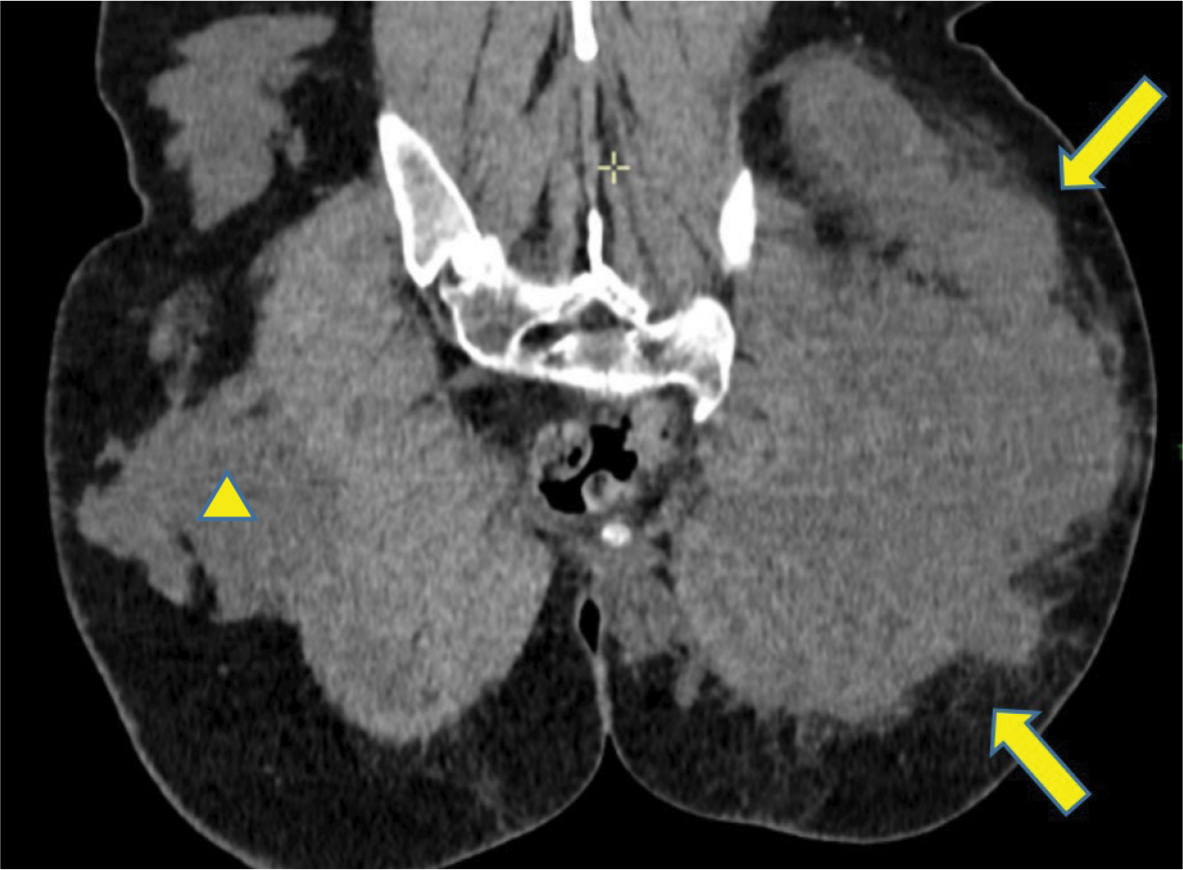
Diffuse infiltration of the subcutaneous fat surrounding the left gluteal fluid collection (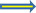).

**Figure 3:**
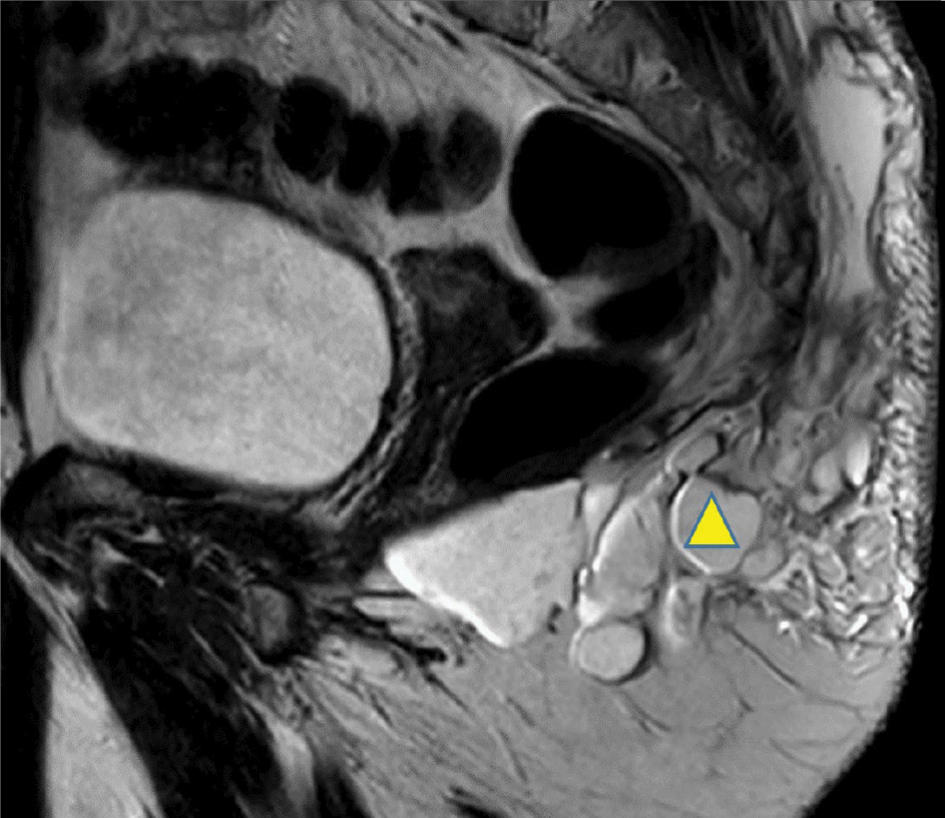
Collection had a multilobulated appearance (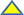).

Given the clinical and biological findings, magnetic resonance imaging (MRI) was performed immediately. The MRI revealed two large fluid multiloculary collections in the subcutaneous gluteal region bilaterally associated with extension in the ischio‑anal fossa and pararectal space (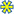; Figure 1). There was moderate diffuse infiltration of the subcutaneous fat surrounding the left gluteal fluid collection (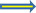; Figure 2). In contrast, the right gluteal collection had a multilobulated appearance (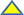; Figure 3), with well‑defined borders and no infiltration of the surrounding fat, corresponding to the normal aspect of the AQUAfilling procedure.

To complement the MRI, a computed tomography (CT) scan was performed to rule out any minor gas component.

## Management

Given the poorly defined nature of the fluid infiltration and its pararectal extension, conservative management with antibiotics (AB) was initiated following a bacteriological sample obtained via interventional radiology. The culture revealed an infection with *Staphylococcus lugdunensis*. The patient was discharged from the hospital against medical advice.

## Discussion

AQUAfilling filler is a hydrophilic gel composed of 98% sodium chloride solution (0.9%) and 2% cation copolyamide, and is described as a sterile synthetic material biocompatible with human tissues. However, this case report perfectly illustrates the long‑term complications expected from AQUAfilling injections, which can lead to multiloculated abscesses, soft tissue inflammation, and migration of the product to more dependent areas [1]. Currently, the toxicity of this material remains uncertain.
